# Autonomic regulation constrains psychotherapeutic workability: a state-dependent translational model for psychotherapy and psychosomatics

**DOI:** 10.3389/fpsyt.2026.1843250

**Published:** 2026-05-11

**Authors:** Eik Niederlohmann

**Affiliations:** Department of Psychosomatic Medicine and Psychotherapy, Kliniken Erlabrunn, Breitenbrunn, Germany

**Keywords:** autonomic regulation, dropout, emotion regulation, interoception, memory updating, non-response, psychosomatics, psychotherapeutic workability

## Abstract

Psychotherapy does not fail only because techniques are poorly chosen or alliances are weak; it may also fail because the patient’s momentary state does not support productive use of the task. This matters especially in psychotherapy and psychosomatic care, where bodily distress, interoceptive threat, shame, and autonomic dysregulation can narrow affective, mnemonic, and reflective workability. In this hypothesis-and-theory paper, I propose a state-dependent translational model of psychotherapeutic processing. Operationally, autonomic regulation is treated not as a binary switch but as a constraint on whether three proposed functional conditions are jointly available in clinically usable form: affective salience, contextual memory access, and reflective working capacity. The amygdala–hippocampus–prefrontal cortex triad is used as a pragmatic shorthand for distributed functions rather than as a one-region explanation of change. The paper integrates emotion-regulation research, autonomic psychophysiology, stress-related prefrontal dysfunction, memory updating, process-based psychotherapy, and selected state-sensitive traditions. It argues that some therapies rely on this logic implicitly, whereas selected state-sensitive traditions—notably ISTDP and related experiential-dynamic approaches—have operationalized real-time state monitoring, titration, and restoration more explicitly. The aim is not to offer a complete neuroscience of psychotherapy, but a clinically teachable and empirically testable model of psychotherapeutic workability relevant to psychosomatic settings, non-response, dropout, and training.

## 1 Introduction: when psychotherapy fails because the patient cannot use it

Psychotherapy non-response and premature dropout remain major clinical and service-level problems. Process research also suggests that psychotherapy unfolds through moment-to-moment microdecisions rather than through techniques acting in a vacuum. One implication is that a treatment may be evidence-based and still become difficult to use in a given session if the patient is underengaged, flooded, or otherwise unable to recruit the functions the task presupposes. In psychosomatic settings, this problem is often especially visible, because bodily threat, shame, interoceptive amplification, pain, dissociation, and autonomic instability are not merely background conditions but active participants in the therapeutic process.

**Figure 1:**
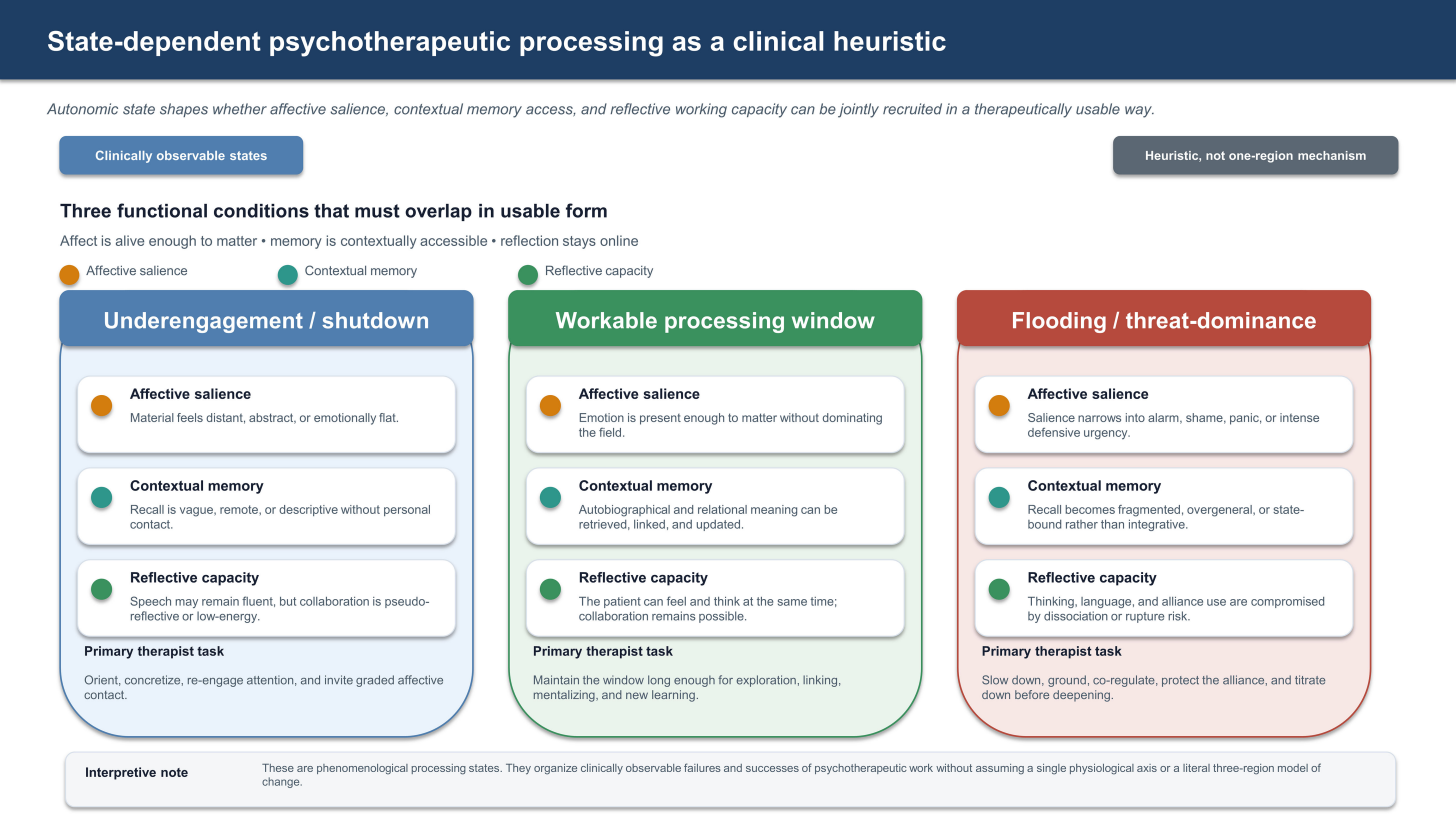
State-dependent psychotherapeutic processing as a clinical heuristic. Autonomic state influences whether affective salience, contextual memory access, and reflective working capacity can be jointly recruited in a therapeutically usable way. The figure organizes clinically observable processing states rather than implying a single physiological axis or a one-region explanation of psychotherapy outcome.

**Figure 2:**
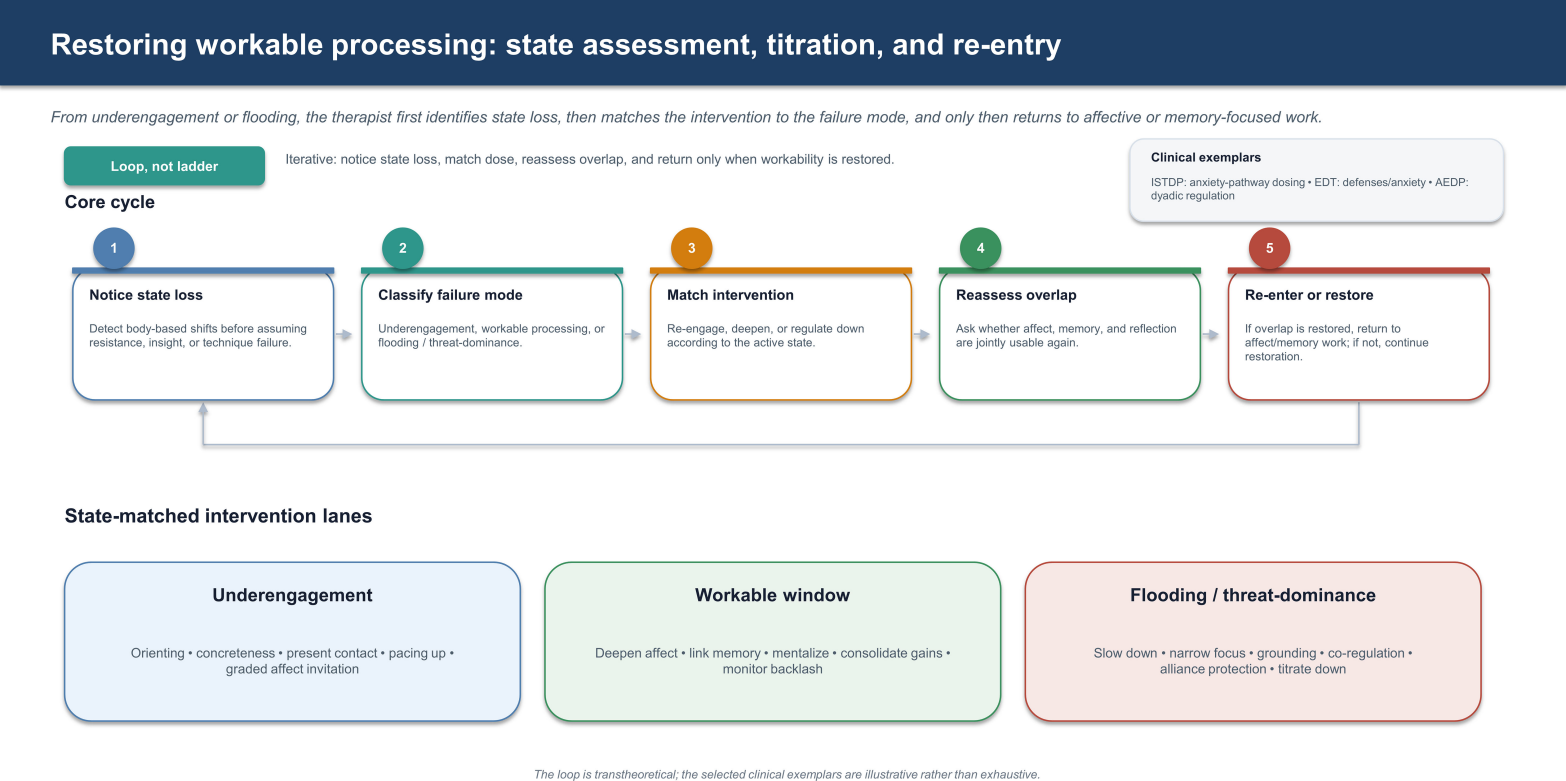
Restoring workable processing: state assessment, titration, and re-entry. From underengagement or flooding, the therapist first identifies state loss, then matches the intervention to the failure mode, and only then returns to affective or memory-focused work. The loop is transtheoretical; the selected clinical exemplars are illustrative rather than exhaustive.

The present paper develops a state-dependent model of psychotherapeutic processing. Its core claim is modest: many psychotherapeutic tasks require a workable overlap of affective salience, contextual memory access, and reflective working capacity. When that overlap is not available in usable form, interpretation, exposure, memory work, or insight-oriented exploration may become less productive, more superficial, or occasionally destabilizing. The central question is therefore not only what intervention is indicated, but whether the patient is in a state that allows the intervention to function as psychotherapy rather than as noise, pressure, or flooding. **Figures 1**, **2** provide a visual overview of the proposed state-dependent processing heuristic and the associated restoration loop.

This framing is not meant to replace existing psychotherapies. Instead, it offers a translational, teachable, and hypothesis-generating model of psychotherapeutic workability with particular relevance for psychotherapy and psychosomatics. It is designed to be school-neutral, while acknowledging that some traditions—especially Intensive Short-Term Dynamic Psychotherapy (ISTDP), broader experiential-dynamic therapy (EDT), and Accelerated Experiential Dynamic Psychotherapy (AEDP)—have already operationalized state monitoring and titrated therapist response more explicitly than many others.

## 2 What this model is—and is not

### 2.1 Level of explanation

The model proposed here is a translational middle-range clinical model. It uses neurobiological shorthand to organize clinically observable functions, but it is not a claim that single brain regions cause psychotherapy outcome in a simple linear way. Throughout this paper, psychotherapeutic workability refers operationally to whether affective salience, contextual memory access, and reflective working capacity are sufficiently co-available, in clinically usable form, for a given intervention at a given moment. References to the amygdala, hippocampus, and prefrontal cortex should therefore be read as compact labels for distributed functions that are repeatedly implicated in emotion, contextual memory, and reflective control, not as a literal three-node theory of psychotherapy.

### 2.2 What the model does not claim

The present model does not claim to be a complete neuroscience of psychotherapy, a universal explanation of all therapeutic change, a new psychotherapy school, or a replacement for alliance, meaning, technique, attachment, learning, culture, or development. It does not assume a single physiological axis of change, and it does not imply that all psychotherapies rely on exactly the same balance of affect, memory, and reflection in every phase of treatment. The key constructs, selected clinical exemplars, adjacent frameworks, and resulting hypotheses are summarized in **Tables 1**–**4**.

**Table 1:** Core constructs of the present model: clinical function, level of explanation, observable markers, candidate measures, and evidential status.

Construct	Clinical function	Level of explanation	Observable markers	Candidate measures	Evidential status
Affective salience	Emotion is present enough to matter without dominating the field	Clinical function with affective-neuroscience shorthand	Flatness, emotional distance, alarm, shame, panic, urgency	Session coding, affect labeling, patient/therapist ratings, physiology proxies	Indirect support from emotion-regulation and stress literatures; clinical synthesis
Contextual memory access	Autobiographical and relational meaning can be retrieved and updated	Clinical function with memory-systems shorthand	Vague, remote, fragmented, overgeneral, or linked recall	Memory specificity tasks, transcript coding, post-session integration markers	Compatible with memory-updating/reconsolidation literature; not a settled necessity claim
Reflective working capacity	Feeling and thinking remain jointly usable in collaboration	Clinical function with executive-control shorthand	Pseudo-reflection, collapse into panic, dissociation, rigid defense, rupture risk	Alliance/process ratings, transcript coding, executive-load tasks, physiology proxies	Indirect support from stress–PFC literature and psychotherapy process research
Workable processing window	Usable overlap of affect, memory, and reflection for therapeutic work	Translational clinical construct	Patient can feel, remember, think, and collaborate at the same time	Integrated session coding, idiographic baselines, multimodal within-person designs	Hypothesis-generating construct proposed in the present paper

The table lists proposed functional conditions and associated indicators; it does not claim that these conditions are already established as necessary in every psychotherapy context.

**Table 2:** Illustrative state-sensitive operations in selected psychotherapy traditions relevant to the present model.

Tradition	State signal monitored	Therapist response when window is lost	How affect/memory work is titrated	Why cited here
ISTDP	Anxiety discharge pathways, defensive barriers, tolerance for complex affect	Shift dose, regulate anxiety, clarify defenses, use graded rather than standard format when needed	Deepening occurs only when anxiety is workable enough for emotional processing	Strongest clinical operational exemplar of explicit state-sensitive dosing
Broader EDT	Affect tolerance, dysregulating defenses, anxiety dysregulation	Regulate anxiety and deactivate dysregulating defenses before experiential deepening	Experiential work is intensified only when affect becomes tolerable and usable	Transdiagnostic experiential-dynamic bridge
AEDP	Dyadic safety, shame, aloneness, transformance markers, relational dysregulation	Undo aloneness, co-regulate, metaprocess, restore safety	Emotion is deepened inside dyadic regulation and relational safety	Attachment-informed co-regulation exemplar

The table is not meant to rank therapies or to imply exhaustiveness; it highlights selected traditions that explicitly formalize state monitoring, titration, and restoration.

**Table 3:** Distinguishing the present model from adjacent frameworks addressing state-dependent psychotherapy.

Framework	Main emphasis	Unit of analysis	What it explains well	What the present model adds
Window of tolerance family	Optimal arousal zone between shutdown and hyperarousal	General regulation state	Clinically intuitive state range	Specifies overlap of affect, memory, and reflection as the relevant psychotherapeutic task conditions
Emotion regulation models	How emotions are modulated across situations and strategies	Regulation strategy and process	Why emotion must be neither absent nor overwhelming	Translates regulation into psychotherapy workability and sequencing
Memory reconsolidation/updating	Change through retrieval and modification of emotionally meaningful memory	Memory process	Why emotionally salient retrieval can matter for change	Adds bodily-state conditions under which memory work may become usable or unusable
Predictive processing/active inference	Prediction, precision, and interoception in behavior and psychopathology	Computational-functional account	Embodied prior expectations and interoceptive constraint	Provides a narrower clinician-facing model that does not depend on accepting a full computational framework
Process-based therapy	Transdiagnostic modifiable processes	Treatment targets and mechanisms	Why processes may matter more than brands	Highlights state-dependent workability as a prerequisite process condition

**Table 4:** Testable hypotheses, candidate measures, suggested designs, and falsification paths derived from the present state-dependent model.

Hypothesis	Prediction	Candidate measures	Suggested design	Falsification path
H1. State-dependent process window	Moderate activation predicts stronger within-session progression than either underengagement or flooding	Session coding, alliance/process ratings, physiology proxies	Naturalistic microprocess study with repeated sessions	Equivalent or superior progression at extreme shutdown/flooding states
H2. Memory work requires gating	Memory-focused interventions are more likely to produce integration when delivered inside a workable window	Transcript coding, memory specificity, post-session destabilization vs integration	Microprocess study linking intervention timing to immediate outcome	Memory-focused work is equally beneficial irrespective of state
H3. Explicit restoration reduces rupture/dropout	Matched regulation before deepening reduces rupture and dropout in high-risk patients	Dropout, rupture-repair coding, attendance, early alliance	Comparative naturalistic or pragmatic trial design	No added benefit of explicit restoration/titration
H4. Within-person calibration beats group norms	Idiographic baselines predict useful state transitions better than generic thresholds	Repeated physiology/process tracking, therapist calibration accuracy	Within-person repeated-measures or N-of-1 enriched design	Group norms outperform idiographic baselines
H5. Explicit state-sensitive traditions show particular relevance in bodily-distress populations	Traditions with explicit state monitoring may show added utility in chronic pain, FND, and persistent physical symptoms	Outcome, dropout, healthcare use, process markers	Comparative effectiveness and moderator analyses	No differential value in high bodily-distress/interoceptive-threat contexts

The literature assembled here is purposive rather than systematic. It privileges frameworks that speak most directly to state-dependent processing and clinical workability; other relevant traditions and competing models are therefore addressed selectively rather than exhaustively. The paper should accordingly be read as a hypothesis-generating integration, not as a definitive review of all state-sensitive psychotherapy theories.

### 2.3 What is novel here

Its incremental contribution is more specific. First, it formalizes state-sensitive workability as a clinically teachable process model. Second, it treats state restoration and titration as explicit therapeutic tasks rather than as background common factors. Third, it makes psychosomatic visibility central: bodily distress, interoceptive amplification, and autonomic dysregulation are not add-ons but contexts in which state-dependent processing becomes especially legible. Finally, it links these claims to operational markers, comparative tables, and falsifiable hypotheses.

## 3 Minimal functional conditions for workable psychotherapeutic processing

Many psychotherapeutic procedures presume that at least three functions are simultaneously available in usable form: enough affective salience for material to matter, enough contextual memory access for experience to be linked and updated, and enough reflective working capacity for collaboration, symbolization, and mentalization. The present manuscript treats these as functional conditions rather than as empirically proven universal necessities. Their usefulness lies in their clinical coherence and testability.

### 3.1 Affective salience: enough emotion to matter, not enough to flood

Emotion regulation research consistently shows that productive psychological work depends on neither affective flatness nor uncontrolled alarm, but on a modulated level of salience in which emotion can be noticed, named, and used (1–3). In psychotherapy, the relevant question is not whether emotion is present, but whether it is present in a way that still allows the task at hand to remain usable.

### 3.2 Contextual memory access: memory must be emotionally present and still transformable

Psychotherapy often requires access to autobiographical, relational, or symptom-relevant memory traces. Emotional memory research and memory-updating accounts suggest that change becomes more plausible when personally meaningful memory can be contacted and revised in a new context (4–6). This does not mean that reconsolidation is the single or final mechanism of psychotherapy. Rather, it provides one compatible lens through which state-sensitive memory work can be understood.

### 3.3 Reflective working capacity: the requirement for thought, language, and collaboration

Stress neuroscience has repeatedly shown that acute threat can reduce higher-order prefrontal functioning and shift behavior toward more reactive, habitual, or defensive modes (7). The extrapolation from stress neuroscience to psychotherapy must remain cautious; however, the inferential bridge is clinically plausible: when reflective working capacity collapses, the patient may still speak, but speech can become pseudo-reflective, dissociated from affect, or unusable for collaborative processing. Psychotherapy needs more than verbal output; it needs workable reflection.

## 4 Body state, clinically observable processing states, and psychosomatic visibility

### 4.1 Why body state comes first

A state-sensitive psychotherapy does not assume that technique failure always reflects resistance, deficient insight, or insufficient courage. Sometimes the task fails because bodily state has already narrowed the processing field. In that sense, body state comes first not because it explains everything, but because it constrains what can be used next.

### 4.2 Underengagement, workable processing, and flooding

Three clinically observable processing states are especially useful. Underengagement or shutdown describes low affective contact, distant or abstract material, and reduced initiative. A workable processing window describes sessions in which affect, memory, and reflection are jointly usable. Flooding or threat-dominance describes states in which alarm, shame, panic, dissociation, or rigid defense compromise memory and collaboration. These are phenomenological process states, not diagnoses. They overlap with the broader family of window-of-tolerance formulations, but the present model narrows the question to psychotherapy use: not simply whether arousal is high or low, but whether affect, contextual memory, and reflection are jointly usable for the next therapeutic task.

### 4.3 Why psychosomatic settings make state shifts especially visible

Psychosomatic and persistent physical symptom presentations make these shifts particularly visible. Bodily distress, pain, functional symptoms, interoceptive amplification, shame, and threat sensitivity often provide an unusually direct view of the moment-to-moment relationship between autonomic state and psychotherapeutic workability (8, 9). In such settings, the difference between processing and pseudo-processing is often clinically obvious: the patient may narrate, but without affective contact; remember, but without contextual linkage; or feel intensely, but without usable reflection.

## 5 State restoration, titration, and explicit clinical exemplars

### 5.1 Why restoring workability deserves its own section

Identifying a non-workable state is insufficient unless the therapist can also respond in a state-matched way. This is one of the most important practical implications of the model. When the window is lost, the immediate task is often not deeper interpretation, not more exposure, and not more memory retrieval, but restoration of workability.

### 5.2 Restoring workability from underengagement

From underengagement, the therapist may need to re-establish contact before asking for depth. Useful operations can include orienting to present experience, increasing concreteness, re-engaging attention, linking distant narration to immediate relevance, and inviting graded affective contact rather than demanding intensity. The aim is not activation for its own sake, but enough salience for the material to become experientially usable.

### 5.3 Restoring workability from flooding

From flooding or threat-dominance, the therapist often has to narrow the field rather than deepen it. Slowing down, grounding, focusing on one channel of experience at a time, protecting the alliance, and co-regulating before returning to memory or affect work are all state-restorative operations. The principle is simple: regulate first, deepen second. Related psychophysiological perspectives on psychotherapy likewise suggest that vagally mediated regulation and social safety processes shape whether deeper therapeutic work remains usable (10, 11).

### 5.4 Intensive short-term dynamic psychotherapy as an explicit operational exemplar

ISTDP is particularly useful as an operational exemplar because it does not merely note that body state matters; it attempts to match therapist action to clinically observable anxiety discharge pathways. Contemporary reviews describe ISTDP as an emotion- and somatically focused brief therapy that explicitly assesses anxiety channeled through striated muscle tension, smooth muscle activation, cognitive-perceptual disruption, and striated weakness (9). In this framework, smooth muscle anxiety or cognitive-perceptual disruption signal that deeper emotional work is currently difficult to use and that anxiety regulation, defensive clarification, or graded dosing should come first.

This makes ISTDP highly relevant to the present model. It provides a concrete example of state-sensitive dosing through graded versus standard formats, explicit anxiety monitoring, and intervention matching. Its empirical literature also links emotional processing to symptom reduction in medically unexplained symptoms, chronic pain, functional neurological symptoms, and broader somatic presentations (12–15). For the present model, ISTDP is therefore most useful not as a replacement framework, but as the clearest existing clinical exemplar of explicit state restoration.

### 5.5 Related experiential-dynamic exemplars: broader EDT and AEDP

Broader experiential-dynamic traditions also support the central logic of the present model. Affect-focused dynamic approaches describe how patients can become frightened of emotion itself and how work must be titrated so that affect becomes tolerable and transformable rather than overwhelming (16). In a more explicitly regulation-oriented formulation, Frederickson and colleagues describe how dysregulated anxiety and dysregulating defenses interfere with emotional processing and how therapy can regulate anxiety before deepening experience (17).

AEDP adds an attachment-informed and dyadic language to the same family of problems. Fosha’s emphasis on undoing aloneness, dyadic regulation, and metaprocessing provides an especially clear example of how affective work is deepened inside a relationally safe and co-regulated field (18). The present model does not claim that ISTDP, EDT, and AEDP are identical. It cites them because they already operationalize the idea that affective work must be dosed in relation to bodily and relational workability. These exemplars are cited illustratively, not as an exhaustive inventory of all compatible state-sensitive psychotherapy traditions.

## 6 Relation to adjacent frameworks and unique contribution

### 6.1 Emotion regulation

The present model is deeply compatible with emotion regulation research. Its distinctive move is not to replace that literature, but to translate it into a psychotherapy-process question: when are affective salience, memory access, and reflection jointly usable for therapeutic work? This keeps the focus on process viability rather than on regulation strategy alone.

### 6.2 Memory updating and reconsolidation

Memory reconsolidation and related memory-updating accounts remain important because they help explain why emotionally meaningful retrieval inside a new context can matter. At the same time, they should be treated cautiously. The present paper therefore uses reconsolidation as one compatible explanatory language, not as the settled or exclusive mechanism of change.

### 6.3 Predictive processing and active inference

Predictive processing and active inference provide a possible meta-language for how bodily threat expectations and interoceptive precision can constrain perception, affect, and action (19–21). For the current model, however, this language is secondary rather than foundational. One does not have to endorse active inference as a unifying theory of psychotherapy to accept the narrower clinical proposition that bodily state can determine whether affective and reflective work remain jointly usable.

### 6.4 Process-based therapy and transtheoretical relevance

Process-based therapy argues that mechanisms and targets may matter more than therapy brands (22). The present model is congruent with that direction. Its specific addition is to suggest that state-sensitive workability is often a prerequisite for process-based work to become clinically usable in the first place.

### 6.5 What this model adds beyond adjacent frameworks

The present model does not outperform these adjacent frameworks by breadth. Its value lies in precision of clinical use: it names a small set of functional overlaps, distinguishes three recurrent observable states, adds explicit state-restoration logic, and shows that some traditions have already operationalized these issues more clearly than others. It is therefore best understood as a narrower clinical workability model rather than a substitute for broader theories of arousal regulation, reconsolidation, alliance, or process-based change.

## 7 Operationalization and research agenda

### 7.1 Core constructs and observable markers

A major advantage of the present model is that it can be operationalized without pretending to be complete. Session coding can assess signs of underengagement, workable overlap, and flooding; therapists and patients can rate task usability; and physiology can be used as a partial, not definitive, proxy. The preferred logic is idiographic and repeated rather than one-off and norm-only.

### 7.2 Testable hypotheses

The model is useful only if it yields hypotheses that can in principle fail when assessed with concrete process, physiological, or outcome indicators. Four particularly important hypotheses concern the existence of a state-dependent process window, the state dependence of memory work, the clinical value of explicit state restoration, and the superiority of within-person calibration over generic thresholds.

### 7.3 Candidate measures and study designs

The model lends itself to multimethod work. Session coding can track state, intervention timing, rupture and repair, and progression.

Physiology can include HRV, electrodermal activity, respiratory patterning, and other autonomic indices, provided they are interpreted cautiously and idiographically. Designs can range from naturalistic repeated-measures process studies to pragmatic comparative trials in chronic pain, functional neurological disorder, and persistent physical symptom services.

### 7.4 Training and implementation research

Because the model is deliberately teachable, it also opens a training agenda. Supervision, deliberate practice, shared microprocess language, and interprofessional psychosomatic team use are plausible implementation targets. A clinically useful model should help people notice state loss sooner, titrate more deliberately, and reduce pseudo-processing.

## 8 Discussion

### 8.1 What this model contributes

The present paper offers a modest contribution: a translational clinical model of psychotherapeutic workability. It does not claim to explain all change, but it clarifies one set of state conditions under which many psychotherapeutic methods become usable, less usable, or temporarily counterproductive.

### 8.2 Clinical implications

Clinically, the model strengthens a simple but often neglected distinction: processing is not the same as talking, feeling, or remembering in isolation. The therapist’s task is not only to deepen work, but to detect when workability has narrowed and to restore it before pushing ahead. This has implications for pacing, sequencing, rupture prevention, and the differentiation of genuine processing from pseudo-processing.

### 8.3 Psychosomatic implications

The model is especially relevant for psychosomatic care because bodily distress makes state shifts harder to ignore. Pain, functional symptoms, panic-like states, interoceptive amplification, and shame often show whether the patient can currently use the task. This may help explain why patients with persistent physical symptoms often oscillate between underengagement, alarm, and fragmented memory while appearing verbally engaged.

### 8.4 Boundary conditions, limitations, and controversies

Several limitations are important. First, autonomic regulation is not the sole determinant of psychotherapy outcome. Technique, alliance, meaning, developmental history, social context, medication, and medical disease all matter. Second, the model uses neurobiological shorthand and therefore should not be read as a literal one-region explanation. Third, reconsolidation and polyvagal literatures remain debated; they are included here as useful but contestable resources rather than as settled foundations. Fourth, an excessive focus on stabilization can itself become a clinical trap if it delays all affective work indefinitely. The model therefore argues for titration, not endless postponement. Finally, the literature synthesis is purposive rather than systematic and may underrepresent other relevant state-sensitive traditions or competing explanations. Other psychotherapy traditions may also contain state-sensitive operations without formalizing them in the same bodily-process vocabulary; they were not included here because the synthesis prioritized approaches that make state monitoring and intervention matching especially explicit. Additional literature on emotion-focused interviewing, short-term psychodynamic approaches to somatic symptoms, active inference, amygdala-prefrontal connectivity, chronic pain trials, deliberate practice, dropout, biopsychosocial theory, alliance, ISTDP mechanisms, psychotherapy microprocesses, polyvagal/neurovisceral integration, and cost-effectiveness provides further background for the proposed state-sensitive framework (23–39).

## 9 Conclusion

Autonomic regulation does not replace psychotherapy. It helps clarify when psychotherapy can function as psychotherapy. By framing workability as a state-dependent overlap of affective salience, contextual memory access, and reflective working capacity—and by making state restoration an explicit clinical task—the present model aims to offer a more teachable, testable, and psychosomatically relevant account of why therapeutic methods sometimes succeed, sometimes flatten, and sometimes flood.

## Data Availability

The original contributions presented in the study are included in the article/supplementary material. Further inquiries can be directed to the corresponding author.
